# A new Late Cretaceous family of Hymenoptera, and phylogeny of the Plumariidae and Chrysidoidea (Aculeata)

**DOI:** 10.3897/zookeys.130.1591

**Published:** 2011-09-24

**Authors:** Denis J. Brothers

**Affiliations:** 1 School of Biological and Conservation Sciences, University of KwaZulu-Natal, Pietermaritzburg, Private Bag X01, Scottsville, 3209 South Africa

**Keywords:** amber, fossil, Plumalexiidae, Plumalexius rasnitsyni, new genus, new species, classification

## Abstract

The taxonomic placement of an enigmatic species of wasp known from two specimens in Late Cretaceous New Jersey amber is investigated through cladistic analyses of 90 morphological characters for 33 terminals ranging across non-Aculeata, non-Chrysidoidea, most subfamilies of Chrysidoidea and all genera of Plumariidae (the family to which the fossils were initially assigned), based on use of exemplars. The fossil taxon is apparently basal in Chrysidoidea, most likely sister to Plumariidae, but perhaps sister to the remaining chrysidoids, or even sister to Chrysidoidea as a whole. It is described as representing a new family, Plumalexiidae **fam. n.**, containing a single species, *Plumalexius rasnitsyni* **gen. et sp. n.** Previous estimates of relationships for the genera of Plumariidae and for the higher taxa of Chrysidoidea are mostly confirmed. The importance of outgroup choice, and additivity and weighting of characters are demonstrated.

## Introduction

**Figures 1–2.**
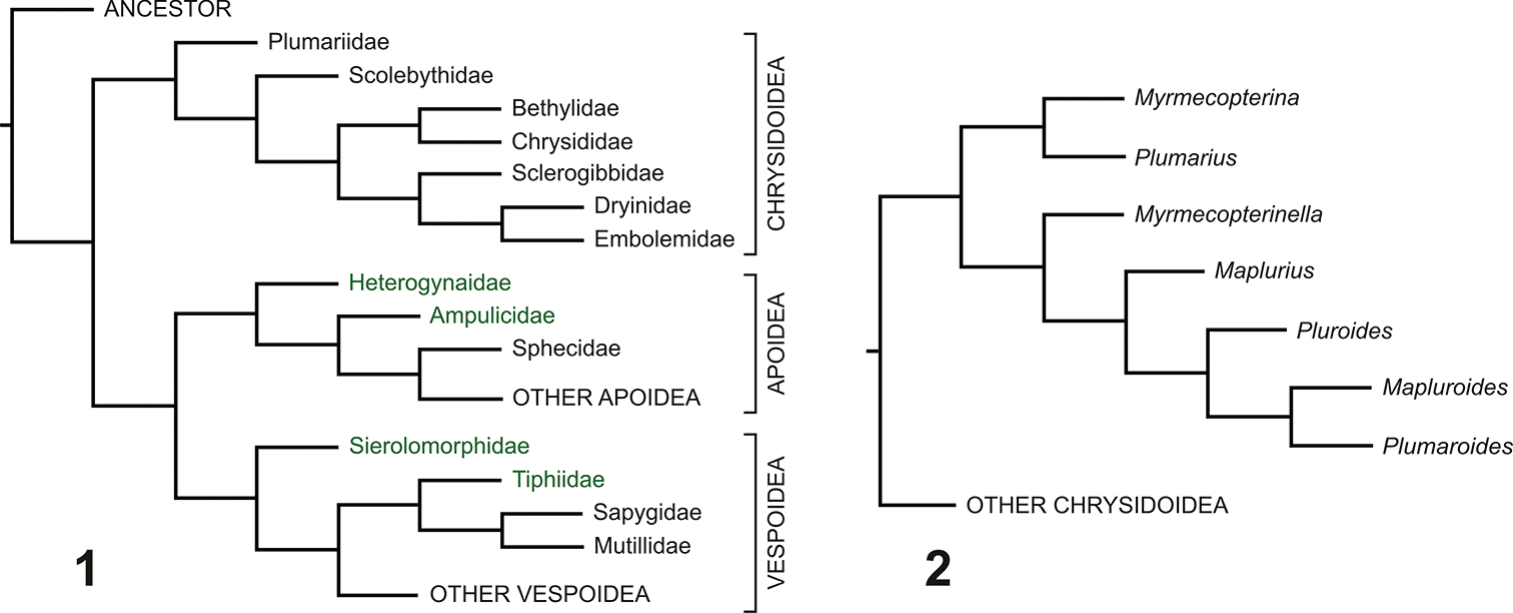
Previous estimates of relationships. **1** Aculeata, superfamilies, and families of Chrysidoidea (modified from Brothers 1999); aculeate taxa used as outgroup for analysis shown in green **2** Genera of Plumariidae (redrawn from Diez, Roig-Alsina and Fidalgo 2010)

The phylogeny of the Hymenoptera, and particularly the Aculeata, has recently been investigated critically by several authors (Brothers 1999; Ronquist 1999; Ronquist et al. 1999 ; Sharkey 2007; Davis, Baldauf and Mayhew 2010; Heraty et al. 2011). Generally, recent authors agree that the Aculeata is monophyletic and comprises two monophyletic lineages, the Chrysidoidea and the (Apoidea + Vespoidea), the latter group sometimes called the “Aculeata sensu stricto (s.str.)”. All also agree that the Plumariidae is the sister group of the remaining Chrysidoidea (see also Carpenter 1999). This paper concentrates on the Chrysidoidea, and the relevant relationships so far established are shown in Figure 1.

As chrysidoids, plumariids are very unusual morphologically, the males having broad wings with a relatively rich wing venation including well developed accessory veins in the apical membrane similar to those of many of the very distantly related Mutillidae (Vespoidea) and Heterogynaidae (Apoidea) (the latter consequently mistakenly assigned to Plumariidae by Brothers 1974), and the females being wingless with the thorax highly modified (in particular with the propleura fused into a tube and a deep ventral constriction at the base of the laterally expanded metathorax-propodeum). No females have been directly associated with males and the correspondence is putative although very strongly supported on distributional and morphological grounds (Evans 1967; Brothers 1984). (The supposed female of *Plumaroides tiphlus* Diez, 2008 is actually a member of the genus *Pseudisobrachium* Kieffer, Bethylidae, according to Quintero and Cambra 2010.) Plumariids occur in the more arid regions of the southern hemisphere, the males often being attracted to lights and the females having been collected under stones or in pitfall traps, but nothing further is known of their biology. There are seven modern genera: *Plumarius* Philippi, 1873, *Plumaroides* Brothers, 1974, *Maplurius* Roig-Alsina, 1994, *Mapluroides* Diez, Fidalgo and Roig-Alsina, 2007 and *Pluroides* Diez, Roig-Alsina and Fidalgo, 2010 from South America, and *Myrmecopterina* Bischoff, 1914 and *Myrmecopterinella* Day, 1977 from southern Africa. Phylogenetic analyses of generic relationships within the family (Roig-Alsina 1994; Carpenter 1999; Diez, Roig-Alsina and Fidalgo 2010) have shown that each southern African genus is most closely related to one or more of the South American genera rather than their being most closely related to each other (Figure 2). The distribution is unusual and putatively Gondwanan, with each of the two primary lineages occurring on both continents and therefore probably having arisen before the breakup of Gondwana. There are no records from the other major Gondwanan continent, Australia, nor from India or the Middle East (other Gondwanan derivatives with arid environments) however.

Within the Chrysidoidea, the family which apparently arose next (after the Plumariidae had diverged) is the Scolebythidae (see Figure 1). This also has a putatively Gondwanan distribution, based on modern representatives, with *Pristapenesia* Brues, 1933 in the neotropics, *Clystopsenella* Kieffer, 1911 in the neotropics and Australia, *Ycaploca* Nagy, 1975 in South Africa, Australia, Fiji, New Zealand and New Caledonia, and *Scolebythus* Evans, 1963 in Madagascar and South Africa (Engel and Grimaldi 2007; CSIRO 2011; Brothers *pers. obs.*); the recent description of a species of *Pristapenesia* from Thailand and China (Oriental and Palaearctic regions) (Azevedo, Xu and Beaver 2011) has cast doubt on the validity of the previous statement, though. Again, the closest relationships are between genera separated by long distances, with *Clystopsenella* and *Scolebythus* being sister groups, as are *Ycaploca* and *Pristapenesia*, as shown by Engel and Grimaldi (2007). However, fossil species of Scolebythidae (including two *Pristapenesia*) have been found in Early Cretaceous Lebanese amber, Late Cretaceous New Jersey amber, Eocene French amber, Eocene Baltic amber and Miocene Dominican amber (Engel and Grimaldi 2007), demonstrating that the family was much more widespread in the past. Strikingly, the fossil members generally are more derived with more reduced wing venation than most modern members. The other (“higher”) families of Chrysidoidea are all also known from fossils, in each case, as for Scolebythidae, the earliest being from the Early Cretaceous (see Engel and Grimaldi 2006; Rasnitsyn 2010; Ortega-Blanco, Delclòs and Engel 2011).

No fossil Plumariidae have yet been described, but two conspecific male specimens from Late Cretaceous New Jersey (USA) amber were recently stated to be members of the family (Rasnitsyn in Grimaldi, Shedrinsky and Wampler 2000; Brothers and Rasnitsyn 2000a,b) and this interpretation has been incorporated in some general accounts (e.g. Rasnitsyn 2002: 244, 2010), although some others (e.g. Grimaldi and Engel 2005: 430; Engel and Grimaldi 2006; Quintero and Cambra 2010) have reflected my subsequent view (Brothers 2003, 2004) that they may represent a new family. The long setae on the flagellomeres, reduced pronotum, large propleura and broad wings with a large pterostigma are particularly reminiscent of Plumariidae, although none of these features is unique to that family. The specimens differ from all modern male plumariids in having much simpler wing venation, lacking the accessory veins and with the second submarginal cell broadly sessile anteriorly, a much smaller anal (vannal/plical) lobe on the hind wing, the clypeus simple, the mandible truncate and four-toothed, the metanotum short and the mesopleuron less swollen. Preliminary analyses using the characters and taxa from Brothers and Carpenter (1993) had indicated their placement in Chrysidoidea (Brothers *pers. obs*.), but a more focused study in the context of the Chrysidoidea as a whole is required to establish their true relationship, and coincidentally confirm or refute existing estimates of the relationships of the chrysidoid families. As reported and discussed below, it is concluded that they represent not only a new genus and species, but are indeed most appropriately allocated to a new family.

## Materials and methods

**Figures 3–7.**
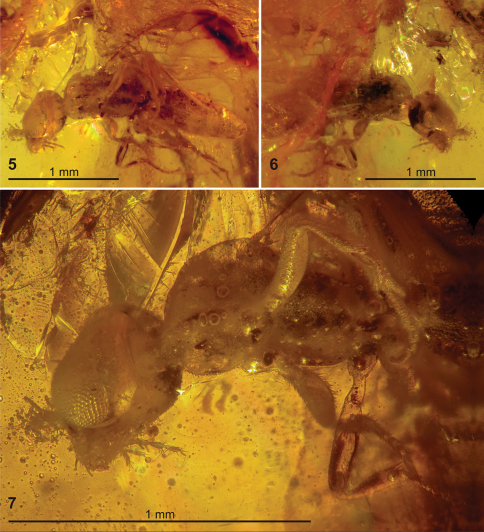
New Jersey amber containing specimens of *Plumalexius rasnitsyni* sp. nov. **3** Specimen NJ-695, holotype (circled) **4** Specimen NJ-175, paratype (circled) **5–7** Specimen NJ-695, holotype **5**  Ventrolateral view **6** Dorsolateral view **7** Detail, ventrolateral view.

The two amber pieces (Figures 3–4), each embedded in epoxy as described by Nascimbene and Silverstein (2000), were studied using standard methods and illustrated using stacked photographs taken with a Canon Powershot G10 digital camera adapted to Wild M7 and Wild M11 microscopes using a Clearshot 600 adapter kit (Alexis Scientific) and combined with CombineZP software (Hadley 2010). Drawings were done using a drawing tube and a Wild M8 microscope and subsequently digitised and corrected with reference to photographs, using CorelDraw X4. As with all fossils, some character states were not absolutely clear, but the most-probable states inferred (as explained in the species description) are those used in the analyses; if such states could not be inferred then they are coded as unknown. The specimens used for the analyses are listed in Appendix A. Terminology has been adapted from previous relevant studies.

Previous cladistic analyses of the Chrysidoidea and Plumariidae (e.g. Brothers, 1999; Carpenter 1999; Diez, Roig Alsina and Hidalgo 2010) have all rooted the trees using hypothetical ancestors with all-primitive states, based on comparisons with either Aculeata s.str. or other chrysidoids, and have utilised groundplans. For this analysis I used exemplars of the various taxa, both outgroup and ingroup, and thus made no a priori assumptions about probable direction of state changes, and thereby also included estimates of polymorphisms. Exemplars rather than groundplans were utilised, as advocated by Prendini (2001), but scorings for individual specimens were often combined to produce “summary” terminals with specified polymorphisms (see Table 1) rather than maintaining them as separate terminals. Since Chrysidoidea is the sister group of Aculeata s.str. (a clade with more-derived states for many characters, as shown by previous analyses), using only members of Aculeata s.str. as outgroups may have been misleading. In addition to other aculeates, I therefore included specimens representing various non-aculeate taxa which have previously been suspected as close relatives of Aculeata (Ichneumonidae, Trigonalidae and Evanioidea). Separate analyses were done using only Aculeata s.str. representatives as outgroup (similar to the approach of Carpenter 1999) and using the expanded outgroup to investigate the influence of using more or less distant taxa as outgroups. For the ingroup, in addition to the two fossil specimens, specimens of all genera of Plumariidae and most subfamilies or (for the smaller families) genera of the other families of Chrysidoidea were examined (Appendix A). In all cases, only males were used since we have no idea what the females of the fossil taxon were like, and there is often considerable sexual dimorphism in chrysidoids and aculeates in general. In a few cases states were derived from or checked in the literature (e.g., Olmi 1984, 1995, 2005; Gauld and Bolton 1988; Kimsey and Bohart 1990; Brothers and Carpenter 1993; Finnamore and Brothers 1993; Huber and Sharkey 1993; Prentice, Poinar and Milki 1996; Brothers and Janzen 1999; Terayama 2003b) specially where the condition of the specimens caused uncertainty, or states were scored as unknown (“?”) for taxa where I had only one or two specimens which could therefore not be dissected. The 90 characters used (see Appendix B) were chosen from those used in previous analyses for plumariid genera, chrysidoid families and aculeates in general, as well as a few newly discovered. The range of characters was thus greater than used in previous analyses.

Parsimony analyses by TNT (Goloboff, Farris and Nixon 2008a, b) were performed using the default settings unless otherwise noted (traditional search, 10 000 replications, tree memory 100 000 trees); implied weighting was implemented using various values of *k* but only those for *k* = 2.5 are reported (this seems to be a reasonable value for the size of the matrix and level of homoplasy found, see Goloboff et al. 2008). Where several most-parsimonious cladograms (MPCs) were found, only the strict consensus is reported. WinClada (Nixon 2002) was used for tree analysis and drawing; branch lengths reflect optimisation of unambiguous states only, with branches unsupported by such states collapsed. In addition to analyses where most characters were considered to be additive (as shown in Appendix B), analyses were also done considering all characters non-additive to investigate the effects of removing all hypotheses of evolutionary direction. Relative group support for all analyses, using GC values which are frequency differences (Goloboff et al. 2003), was estimated by symmetric resampling using TNT (new technology search using ratchet, drift and tree fusing, 10 000 replications, tree memory 100 000 trees).

## Systematic palaeontology

### Family Plumalexiidae

Brothers fam. n.

urn:lsid:zoobank.org:act:621025A6-DE9F-445E-8D8B-05194B6EECF7

http://species-id.net/wiki/Plumalexiidae

#### Type genus.

*Plumalexius* Brothers, new genus.

#### Diagnosis.

Male. Pronotum forming a short convex band reaching tegula; propleura closely associated, anterodorsally exposed as a short neck, posteriorly swollen and transversely truncate; prosternum short and scarcely exposed medially; mesopleuron large and swollen; metasternum somewhat depressed. Forewing with pterostigma very large, seven closed cells (costal, basal, subbasal, marginal, first and second submarginals, first discal), second submarginal cell with long anterior margin, no accessory veins in apical membrane. Hind wing with closed cells (basal and subbasal at least), anal (vannal/plical) lobe well developed; jugal lobe absent. Coxae subglobose, trochanters inserted apically.

Female. Unknown.

### Genus Plumalexius

Brothers gen. n.

urn:lsid:zoobank.org:act:80D51C18-95CB-435D-9794-0CBB59936091

http://species-id.net/wiki/Plumalexius

#### Type species:

*Plumalexius rasnitsyni* Brothers, new species

#### Etymology:

The genus name, which is masculine, is derived from “Plumariidae”, to which it was first assigned, and “Alexandr”, the first name of Professor Dr Rasnitsyn, honoured in this Festschrift.

#### Diagnosis:

Male. Compound eye oval with convex inner margin; antenna with many long fine erect setae (number of antennomeres unknown); mandible with four apical teeth along truncate apical margin; maxillary palp at least 5-segmented; labial palp at least 3-segmented. Pronotum much shorter than mesoscutum, with slight anterior collar (flange) and posteroventral angle rounded; mesoscutum transverse; notaulus distinct, complete; tegula small, convex; metapostnotum apparently about as long as metanotum; propodeum long, weakly constricted apically; meso-metapleural suture straight. Hind wing with two closed cells, vein C present only basally, anal (vannal/plical) lobe less than half length of wing. Tibiae without spines or strong setae; tibial spurs 1–2–2; basitarsomeres much longer than other tarsomeres; arolia large; claws simple. Metasoma ovoid, sessile basally, apical tergum apparently simple, seventh sternum reduced, hypopygium simple with convex apex.

Female. Unknown.

### Plumalexius rasnitsyni

Brothers sp. n.

urn:lsid:zoobank.org:act:20DEDD72-2F53-43D6-B455-E987604C0265

http://species-id.net/wiki/Plumalexius_rasnitsyni

Figs 3 [Fig F3] [Fig F4] –13

#### Type material:

**Figures 8–9. F3:**
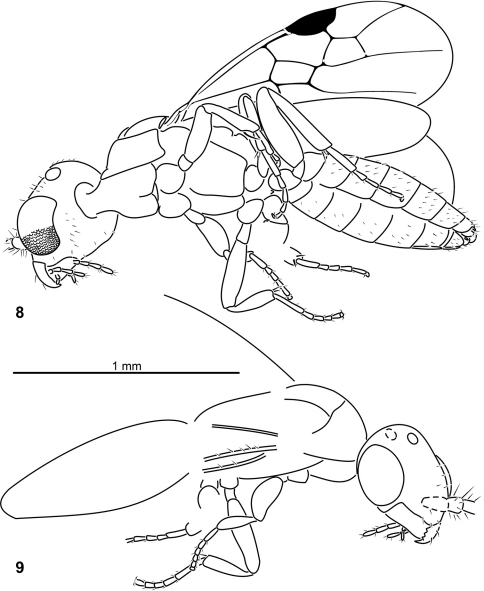
*Plumalexius rasnitsyni* sp. nov., holotype. **8** Ventrolateral view **9** Dorsolateral view.

Holotype male (Figures 3, 5–9), in heavily fractured block of yellowish amber embedded in a trapezoidal epoxy matrix about 22 × 10 × 7 mm, with labels as follows: “NEW JERSEY Amber: / Late Cretaceous / NEW JERSEY: Middlesex Co / Sayreville, White Oaks Pit / 1995, coll.Paul Nascimbene / AMNH no. NJ-695”, “NEW JERSEY Amber: / Late Cretaceous / AMNH no. NJ-695 / HYMENOPTERA:”, “Plumariidae” [Rasnitsyn’s handwriting], “HOLOTYPE / Plumalexius / rasnitsyni ♂ / D.J. Brothers, 2011” [red label, printed].

**Figures 10–11. F4:**
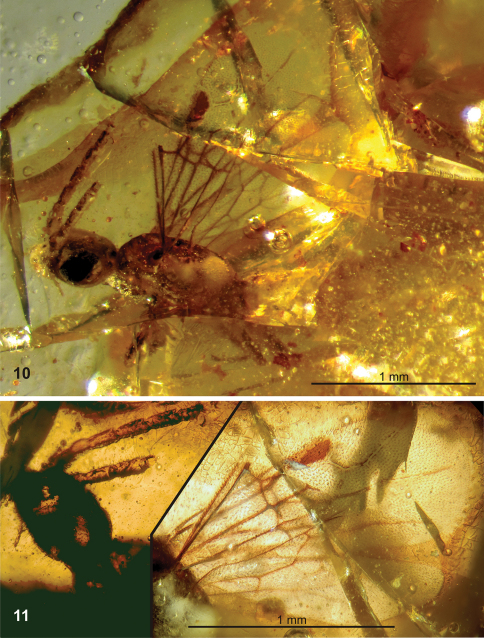
*Plumalexius rasnitsyni* sp. nov., specimen NJ-175, paratype. **10** Dorsolateral view **11** Details, dorsolateral view.

**Figures 12–13.**
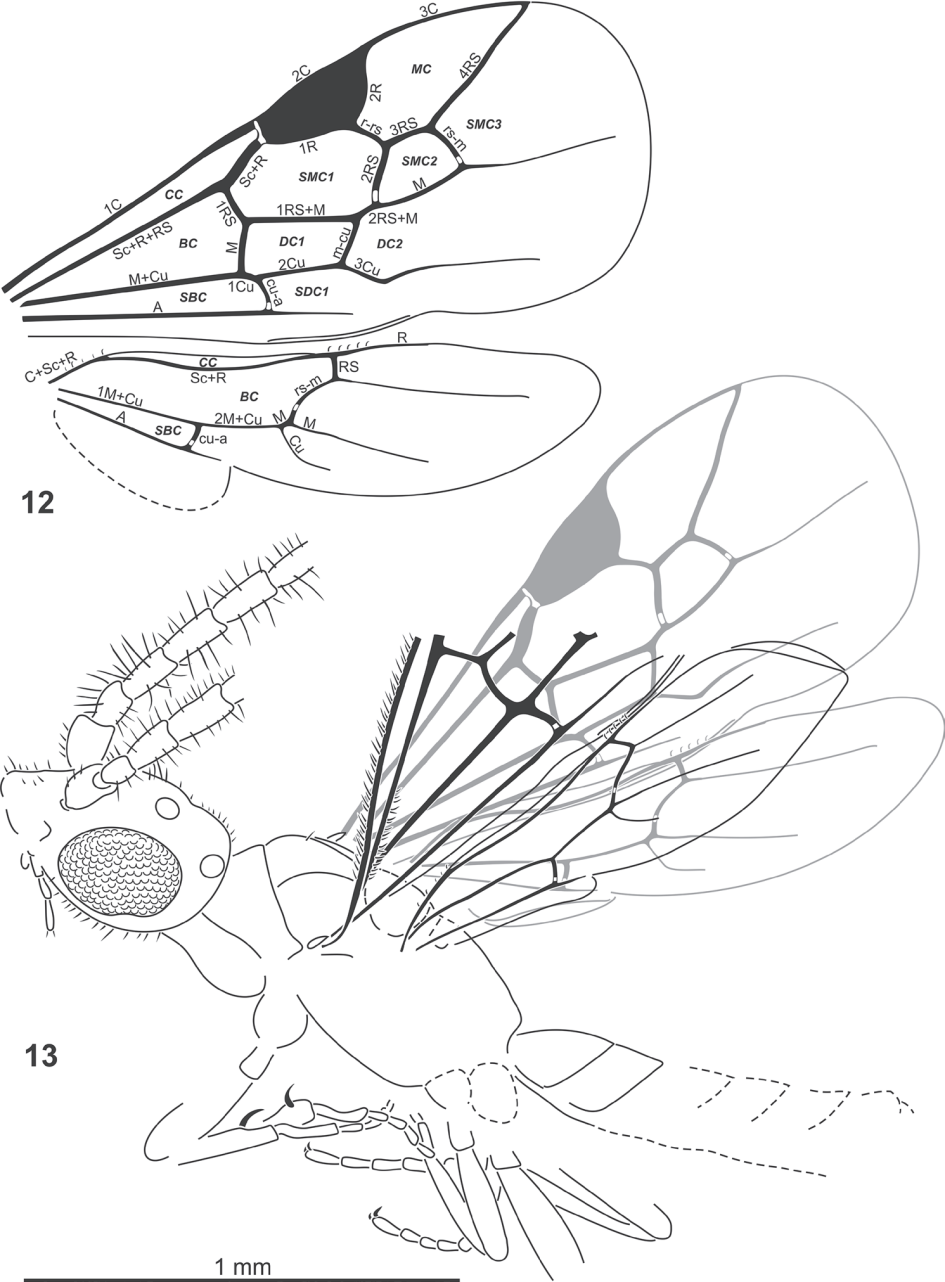
*Plumalexius rasnitsyni* sp. nov. **12** Wings, based on both specimens **13** Paratype, dorsolateral view, right wings in grey. Abbreviations. Wing veins: A = anal, C = costa, Cu = cubitus, M = media, R = radius, RS = radial sector, Sc = subcosta (numerals indicate abscissae, all lower-case indicates crossveins); cells: *BC* = basal cell (cell R), *CC* = costal cell (cell C), *DC* = discal cell (cells 1M, 2M), *MC* = marginal cell (cell 2R1), *SBC* = subbasal cell (cell 1Cu), *SDC* = subdiscal cell (cell 2Cu), *SMC* = submarginal cell (cells 1R1, 1Rs, 2Rs).

Paratype male (Figures 4, 10–13), in heavily fractured block of yellowish amber embedded in a rectangular epoxy matrix about 18.5 × 13.5 × 9 mm, with labels as follows: “NEW JERSEY Amber: / Late Cretaceous / NEW JERSEY: Middlesex Co / Sayreville, White Oaks Pit / 1995, coll.Paul Nascimbene / AMNH no. NJ-175”, “NEW JERSEY Amber: / Late Cretaceous / AMNH no. NJ-175 / HYMENOPTERA: / Family? (PN-2a) / Plumariidae” [Rasnitsyn’s handwriting], “?Family / Det. L. Masner 1996”, “PARATYPE / Plumalexius / rasnitsyni ♂ / D.J. Brothers, 2011” [yellow label, printed]. (This specimen is presumed to be a male because of its similarity to the holotype even though the metasoma is mostly not visible.)

#### Etymology:

The species name, a noun in the genitive case, honours Professor Dr Alexandr Rasnitsyn, who first recognised the significance of the specimens.

#### Description

(based on holotype, paratype data in parentheses where different or feature not visible in holotype): **Male.** Entirely pale yellowish (reddish) brown with venation slightly darker. Head and body length as preserved 2.03 (2.37) mm; estimated head length 0.24 (0.29) mm; estimated mesosoma length 0.80 (0.77) mm; estimated metasoma length 0.90 (0.89) mm; approximate forewing length 1.32 (1.46) mm; approximate hindwing length 1.03 (1.17) mm. Head and metasoma with scattered fine short erect setae, mesosoma almost glabrous, antennal pedicel and flagellomeres with fine long erect setae; legs with dense recumbent setae and scattered semi-erect setae.

Head: Hypognathous; about as wide as high; vertex evenly rounded. Eye ovate with convex inner margin, moderately protuberant, apparently glabrous, ommatidia distinct. Ocelli ovate, large. Occipital carina distinct. Frons and clypeus weakly convex; clypeus transverse with convex anterior/apical margin. Gena simple. Antennal sockets simple, apparently about as close to eyes as to each other, apparently close to posterior/dorsal margin of clypeus. Antennal scape about as long as wide (distinctly flattened posterolaterally and broadened towards apex), with several erect setae; pedicel (about half length of scape and of first flagellomere), with many fine long erect setae; (flagellomeres 1–4 becoming slightly longer sequentially, with many fine long erect setae). Mandible long, evenly broad and curved; two prominent short curved setae on lateral surface; apex truncate with four similar sharp teeth, apical tooth the longest. Maxillary palp at least 5-segmented; labial palp at least 3-segmented [palp bases concealed by foam but segmentation inferred from assumed points of origin].

Mesosoma ovate, about twice as long as wide/high. Pronotum forming a curved oblique ribbon anterolateral to mesoscutum, broader medially than posterolaterally; posterodorsal margin evenly concave; posterolateral margin strongly emarginate and approaching tegula dorsally; posteroventral angle broadly rounded; anteroventrally with slight collar (flange) but leaving propleura exposed anteriorly. Propleura closely associated or fused; anteriorly produced as a short neck; swollen posteriorly; posterior margin apparently almost straight but exposing small part of prosternum medially; forecoxae approximated. Mesoscutum shorter than wide, moderately convex; notaulus distinct and complete, weakly diverging anteriorly; tegula small and convex. (Mesoscutellum apparently almost as long as scutum, weakly convex.) Mesopleuron large and convex; meso-metapleural suture distinct, almost straight. Mesosternum with posteromedial margin almost straight and apparently slightly overhanging mesocoxal base; mesocoxae slightly separated. (Metanotum short and transverse, flattened, not constricted medially. Metapostnotum apparently as long as metanotum, slightly depressed.) Metasternum apparently somewhat depressed. Metacoxae slightly separated. Propodeum slightly longer than mesoscutum, weakly convex but more strongly so posteriorly although without any defined posterior declivity; incision between mesosoma and metasoma weak.

Forewing broad, about 2.2 × as long as wide, about 1.7 (1.8) × as long as mesosoma, with seven closed cells, veins approaching but not reaching margin. Costal cell well developed, broad. Pterostigma large, about 0.19 × as long and 0.17 (0.20) × as wide as wing, entirely sclerotised. Marginal cell about 2.38 (2.17) × as long as wide, 1.41 (1.52) × as long as pterostigma, apex acute. First submarginal cell about 2.08 (2.61) × as long as wide, 0.81 (0.80) × as long as marginal cell. Second submarginal cell almost as large as pterostigma, broadly sessile anteriorly, about 0.64 (0.57) × as long as first submarginal cell. Veins tubular except for nebulous free apical sections of M, Cu and A. No trace of any accessory vein(s) in apical membrane. Prestigmal vein (Sc+R) scarcely swollen, about 1.33 (1.37) × as long as vein 1Rs. Crossvein cu-a distinctly postfurcal, about 1.33 (1.75) × as long as 1Cu. Vein Cu2 absent, first subdiscal cell broadly open apically.

Hind wing about 0.8 × as long as forewing. (Basal and subbasal cells closed by tubular veins; costal cell open anteriorly, vein C present only basally. Veins tubular except for nebulous free apical sections of Rs, M, Cu and A. A few basal hamuli present in a cluster; about five apical hamuli. Crossvein rs-m long, about 2.67 × as long as 1Rs. Vein 1M very short, about 0.13 × as long as 2M+Cu. Crossvein cu-a distantly antefurcal, 2M+Cu about 0.57 × as long as 1M+Cu. Anal (vannal/plical) lobe apically delimited by moderate incision, lobe about 1.19 × as long as submedian cell, about 0.4 × as long as wing.) Jugal lobe absent.

Legs well developed, moderate in size; trochanters well developed and cylindrical; no trochantelli; tibiae without any spines or strong setae; basitarsomeres long, about as long as next three tarsomeres combined; all arolia large and flattened; claws simple ventrally. Foreleg with coxa subglobose, trochanter inserted apically; femur slightly swollen, with inner/anterior surface flattened; tibia with simple, weakly curved, bladelike calcar subapically. Mid- and hind legs with coxae globose, hind coxa somewhat larger than mid-coxa; tibiae each with two straight simple apical spurs, inner spur somewhat longer than outer.

Metasoma elongate oval, about 2.6 (indeterminable in paratype) × as long as wide/high; terga subequal in length. First tergum broad, weakly contracted toward base, profile evenly merging with second. First sternum apparently simple and evenly overlapping second. Seventh tergum apparently simple with apical margin convex. Seventh sternum apparently reduced and mostly concealed. Hypopygium simple, weakly convex, with narrowly rounded apical margin. Cercus apparently present, cylindrical. Genitalia with paramere apparently broadly rounded apically.

**Female.** Unknown.

## Results and discussion

Table 1 shows the distribution of character states across the taxa.

**Figures 14–17.**
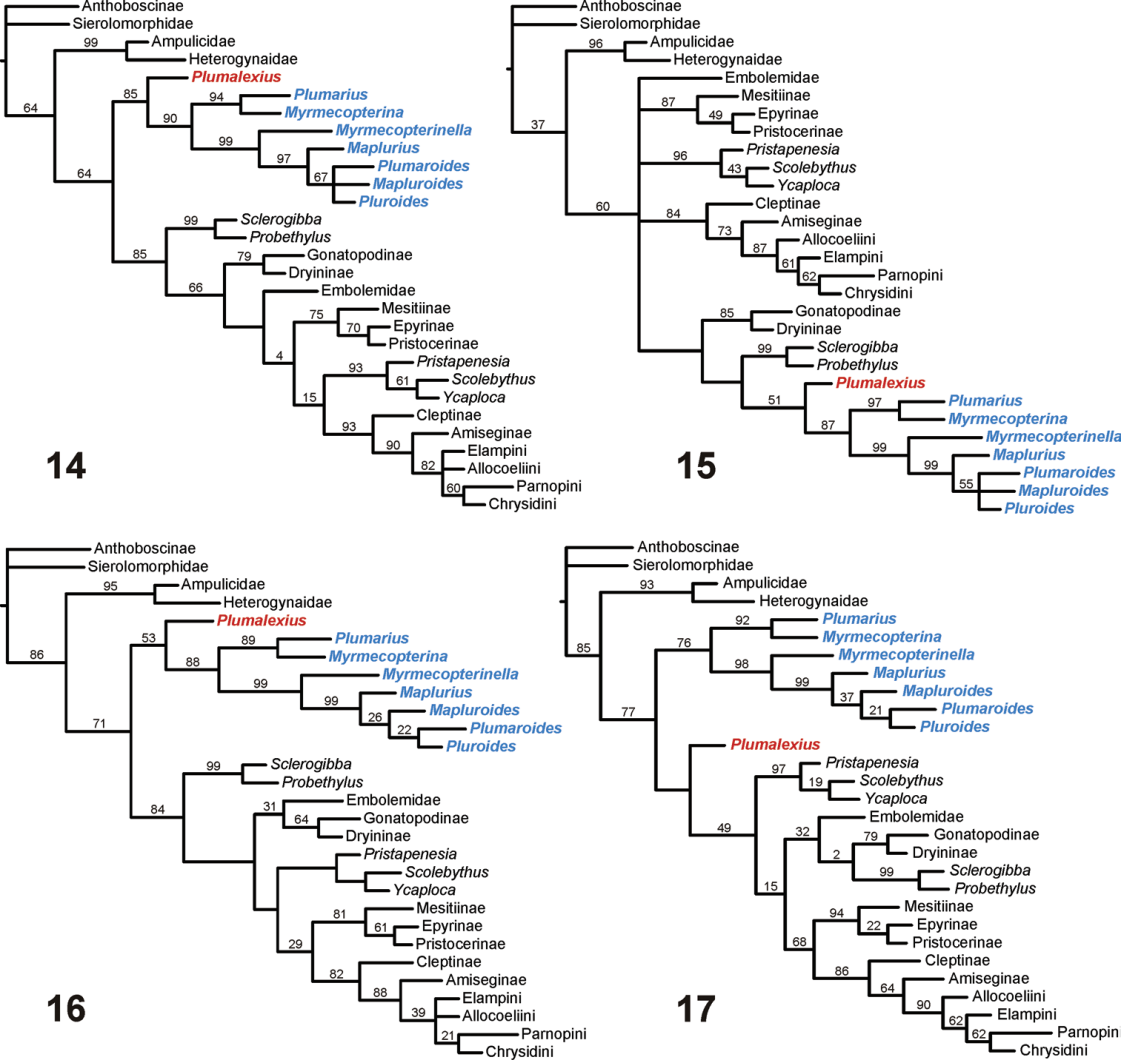
Chrysidoidea relationships using Aculeata s.str.(Anthoboscinae) as outgroup **14** Characters equally weighted, some characters additive (strict consensus of 4 MPCs, raw lengths 383, CI = 0.41, RI = 0.71) **15** Characters equally weighted, all characters non-additive (strict consensus of 4 MPCs, raw lengths 349, CI = 0.45, RI = 0.71) **16** Characters implicitly weighted (*k* = 2.5), some characters additive (strict consensus of 2 MPCs, raw lengths 386, CI = 0.41, RI = 0.71) **17** Characters implicitly weighted (*k* = 2.5), all characters non-additive (1 MPC, raw length 352, CI = 0.44, RI = 0.70). Notes: *Plumalexius* shown in red, genera of Plumariidae shown in blue. Numbers are estimated GC branch-support values (see text); branches without numbers showed no positive support under the resampling protocol used.

The cladograms resulting from the analyses using an aculeate outgroup (illustrated using Anthoboscinae, but the relationships within the Chrysidoidea were not affected by changing this to any of the other three aculeates) are shown in Figures 14–17. The consensus tree from the “equally weighted additive” analysis (Figure 14) shows Chrysidoidea as monophyletic, all chrysidoid families also as monophyletic (although with their relationships often not convincingly resolved, as shown by several apparent clades having no or very low relative resampling support), *Plumalexius* sister to Plumariidae (this clade sister to the remaining chrysidoids), and the plumariid genera with similar relationships to those found earlier (see Figure 2). In contrast, although the consensus tree from the “equally weighted non-additive” analysis (Figure 15) also shows Chrysidoidea as monophyletic, all chrysidoid families also as monophyletic (with their relationships even less resolved), and the same relationships for the plumariid genera, (*Plumalexius* + Plumariidae) now groups with Sclerogibbidae and Dryinidae, although without relative support. The consensus tree from the “implicitly weighted additive” analysis (Figure 16) shows similar relationships as the equally weighted version (Figure 14), except that there is slightly greater resolution for the families of Chrysidoidea and Scolebythidae is no longer sister to Chrysididae which is now monophyletic with Bethylidae, although some branches lack positive relative support values; there is also greater resolution for the plumariid genera. Similarly, the single MPC from the “implicitly weighted non-additive” analysis (Figure 17) produced improved resolution, showing (Bethylidae + Chrysididae) as monophyletic, but *Plumalexius* is grouped with the chrysidoids other than Plumariidae (although without positive relative branch support). The differences from previous analyses, most strikingly involving Scolebythidae and Sclerogibbidae (see Figure 1), are probably due to two factors: the use of exemplars instead of groundplans (introducing polymorphisms), and the position of the Aculeata s.str. outgroup taxa as relatively more derived than the Chrysidoidea.

**Table 1.** Data matrix for analyses of relationships of members of Chrysidoidea. (Specimens used shown in Appendix A, characters in Appendix B. Values between square brackets indicate polymorphisms.)

**Characters**	**1 - 10**	**11 - 20**	**21 - 30**	**31 - 40**	**41 - 50**	**51 - 60**	**61 - 70**	**71 - 80**	**81 - 90**
**Taxa**									
*Ichneumonidae	010100000[012]	000021000[01]	001[01]000202	000[01]1000[03]0	001[01]21[01]00[12]	[45]02?00010[12]	0[34]00000100	002001000[02]	0000000000
*Trigonalidae	1100010012	0000[01]01000	0010000202	0011100030	1000201001	000000000[01]	0301000100	0000000000	0000022200
*Evaniidae	0001100100	0000000001	0010100202	0011101033	5021011001	200-001103	1402100000	00000[01]0000	1000001020
*Gasteruptiidae	0211000100	0000100101	1100000202	0011211033	0011011001	620-000103	1412100100	0001000000	1000001020
Anthoboscinae	0110001112	0001200000	1002100100	1002100030	3300000001	0000000000	0400010000	0001020000	0000121001
Sierolomorphidae	111001111[02]	0001100000	1002100100	0001200012	3112000010	100-001001	1401000100	0011020000	0000002301
Ampulicidae	[01]110000110	0001200001	0000100011	1011120043	221[12]0101[01]1	0000000000	[01][04]01000[01]00	0[01]2[23]0[12]0004	010002[12]021
Heterogynaidae	0110000100	0001200001	0000100011	2011121043	2211010111	300-110100	1101010100	0023000004	0000020221
*Plumalexius*	1??0000?02	20100??100	1?0?1?0???	00001?001?	????1?0010	6100000011	1401010110	1023000000	0??001000?
*Plumarius*	0001100111	2131101101	1100100000	0000100111	4412211000	6101010011	0001010100	002201[01][01]03	0100010000
*Plumaroides*	0000011102	0001112220	1001111200	1000100120	3422211000	6101111010	1001011110	1123020000	011211100?
*Myrmecopterina*	0[12]10100111	2011101101	1100100000	0000100102	44[12][12]211000	6102110011	1001010110	1022011003	0101011000
*Myrmecopterinella*	0000001202	2001213200	0001111200	11001?1132	?411211010	620-011011	1301010110	0013220000	010001010?
*Maplurius*	0000011102	2121113220	1001111200	1000100120	3412211010	6101011011	1001011110	1122020010	010111020?
*Mapluroides*	0000011102	0001112220	1001121200	10000?0120	?411211000	6101011012	0001011110	1122020000	010111020?
*Pluroides*	0000011102	0001113210	1001111200	10001?0120	?411211000	6101011011	0001011110	1122020000	010211100?
*Scolebythus*	1110000100	2000000100	0020001000	1000101020	3301000011	620-001012	2222110111	0002000000	0100021020
*Pristapenesia*	1??0000100	0000[02]00100	00200010??	[12]0001?10??	?322000111	65[01]-00101?	2222110111	0002100000	0100010020
*Ycaploca*	1110000100	1000000100	0020001000	1000101020	3312000010	620-001012	2222110111	0012100000	0100021020
Mesitiinae	1130000122	20[01]0001000	0000100010	0011011032	4122010111	651-000012	2522110100	0021000004	0100020210
Epyrinae	111000012[02]	0000001000	0000100000	1011011032	4122010111	651-001111	2522110100	0021020004	0100010010
Pristocerinae	1[01][012]0000120	[02]0[01]0[02]01000	0000100000	101101[01]032	4122010111	651-001111	2522110100	0021020004	0100020210
Cleptinae	1000000100	0000111000	1010001000	0011110133	3122001111	651-001111	2211110100	0012000004	0100022020
Amiseginae	1110001100	0000221000	1010000010	0011210[01]33	4122001011	651-001011	1222110100	0013000003	0100022020
Elampini	1110001100	0001011001	1010000000	00111?0033	?222001011	65[01]-001110	1322110100	0013000004	0100022??0
Allocoeliini	111000?100	0001211001	1010000000	00111?0033	?222001011	651-001110	1222110100	0013000001	0?00022??0
Parnopini	111000110[02]	0001244001	1010001000	00111?0033	?[02]22001012	651-000112	1322110110	0113010004	0100022??0
Chrysidini	1110001100	0001211001	1010000000	0011[12]10[01]33	4[02]12001011	651-00111[013]	13[12][12]110110	0013000004	0100022020
*Sclerogibba*	0220001020	1001111100	1000100010	0011100022	5121110012	630-000012	2223110100	1022010002	0100010010
*Probethylus*	0220001020	1001132100	1000110010	0011100012	5121110012	630-000012	2223110100	1022010002	0100010010
Gonatopodinae	1220001310	10001[02][12]201	0010000000	0001100132	5022100010	650-001112	1423110110	1022100002	0100022020
Dryininae	1[12][12]0000310	1000101[12]01	0000100000	0011100132	5022[01]00010	650-001112	2523110100	1022100002	0100022020
Embolemidae	[01]1[12]1000300	[01]0000[123][12]001	0000120012	0011101[01]32	5[23]12100111	6[45][01]-00[01][01]1[012]	1423110110	0022000002	01000[12]1020

*Non-aculeate outgroup taxa excluded from some analyses (see text)

**Figures 18–21.**
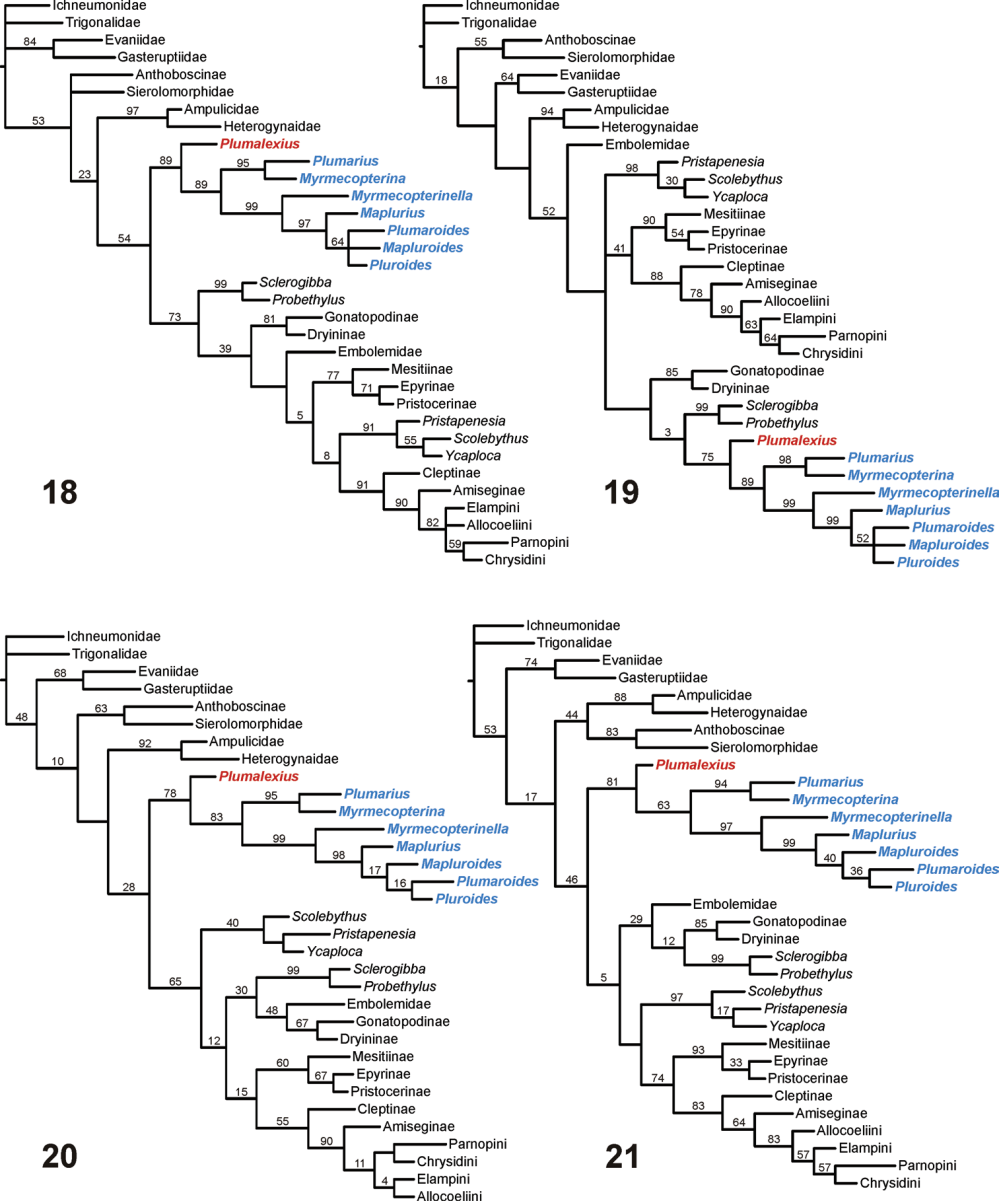
Chrysidoidea relationships using non-Aculeata (Ichneumonidae) as outgroup **18** Characters equally weighted, some characters additive (strict consensus of 12 MPCs, raw lengths 468, CI = 0.35, RI = 0.68) **19** Characters equally weighted, all characters non-additive (strict consensus of 4 MPCs, raw lengths 423, CI = 0.39, RI = 0.67) **20** Characters implicitly weighted (*k* = 2.5), some characters additive (1 MPC, raw length 473, CI = 0.35, RI = 0.68) **21** Characters implicitly weighted (*k* = 2.5), all characters non-additive (1 MPC, raw length 426, CI = 0.38, RI = 0.67). Note: *Plumalexius* shown in red, genera of Plumariidae shown in blue. Numbers are estimated GC branch-support values (see text); branches without numbers showed no positive support under the resampling protocol used.

To try to address the second of the above concerns, analyses were done using four non-Aculeata as outgroup. The resulting cladograms (Figures 18–21) are presented with Ichneumonidae as outgroup, but using any of the other three non-aculeates made no difference to the relationships shown for the Chrysidoidea. The consensus tree from the “equally weighted additive” analysis (Figure 18) shows Chrysidoidea as monophyletic, all chrysidoid families also as monophyletic (but their relationships still sometimes unconventional), *Plumalexius* basal to Plumariidae (with high support), and the plumariid genera with similar relationships to those found earlier. Although the study was not intended to reflect the relationships amongst the outgroup taxa, it is interesting that Aculeata s.str. (Vespoidea and Apoidea) appears as paraphyletic in this analysis. The consensus tree from the “equally weighted non-additive” analysis (Figure 19) also shows Chrysidoidea as monophyletic (but with Embolemidae as basal), all chrysidoid families also as monophyletic but with most relationships very different from previous findings (except that Bethylidae and Chrysididae are well supported as monophyletic, and many of the family relationships have no positive relative branch support), with Plumariidae (and *Plumalexius* sister to it) appearing as most closely related to Sclerogibbidae and Dryinidae; the relationships for the plumariid genera remain consistent. Aculeata s.str. now appears as polyphyletic, with Evanioidea interpolated between Vespoidea and Apoidea. The single MPC resulting from the “implicitly weighted additive” analysis (Figure 20) is fully resolved, shows Chrysidoidea as monophyletic, all chrysidoid families as monophyletic, Plumariidae (and *Plumalexius* sister to it) as sister to the remaining chrysidoids, and the relationships of those families as found by previous analyses (see Figure 1); the relationships of the plumariid genera also agree with previous analyses (see Figure 2), except that *Plumaroides* appears as sister to *Pluroides* rather than *Mapluroides*. Aculeata s.str. is paraphyletic but with the apparent sister-group relationship of Apoidea to Chrysidoidea not supported (the resampling analysis instead showed a monophyletic Aculeata s.str. as supported with a value of 12). The “implicitly weighted non-additive” analysis also produced a single MPC (Figure 21) with a monophyletic Chrysidoidea, monophyletic chrysidoid families, *Plumalexius* sister to Plumariidae, and Bethylidae and Chrysididae forming a monophyletic group but apparently sister to Scolebythidae (but without relative branch support); relationships for the plumariid genera are the same as for the “additive” analysis. Aculeata s.str. is now monophyletic (with good support) and sister to Chrysidoidea (with some positive support). It is notable that the chrysidoid relationships shown are more similar to those for the “additive” analyses than those seen in the “unweighted non-additive” analysis.

**Figure 22.**
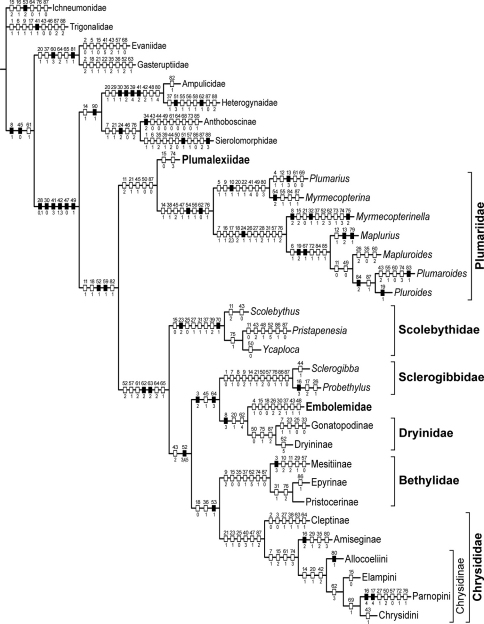
Preferred cladogram of families of Chrysidoidea and genera of Plumariidae (raw length 474, CI = 0.35, RI = 0.68) showing only unambiguous character-state changes. Notes: open hashmarks indicate homoplasious states, black hatchmarks indicate unique states; character numbers above, state numbers below (polymorphisms separated by commas).

At first sight, consideration of all of the above results, involving not only the placement of *Plumalexius* but even more the relationships amongst the other chrysidoids, has produced a slightly confused picture, perhaps not unexpected for a set of analyses using exemplars and considerable polymorphism, and also based on characters which have previously been used at very different levels. The limitation of having to exclude all characters restricted to females (many of which have proved extremely informative in previous analyses, and one of which, the presence of an articulation within gonocoxite IX, is probably the most significant unique synapomorphy for Chrysidoidea) has also had an effect. Nevertheless, it is gratifying that the results of most previous studies have been confirmed, or at least not convincingly contradicted. Accordingly, I consider that the cladogram which agrees best with those results, one using an expanded outgroup and additive characters, and derived using implied weighting (an approach advocated by Goloboff et al. 2008), should be considered the preferred current estimate of the relationships of the families of Chrysidoidea and the genera of Plumariidae. This is shown in Figure 22, elaborated and adjusted from Figure 20, with Aculeata s.str. shown as monophyletic (which increased the length of the tree by a single step) and the relationships of the tribes of Chrysidinae resolved to reflect that found (and supported) most often in all analyses (which did not alter the tree length). It must be noted that, although for each family some subfamilies, tribes and genera are also shown, and their apparent relationships often (but not always) agree with other recent studies (such as Carpenter 1999; Terayama 2003a; Engel and Grimaldi 2007; Carr, Young and Mayhew 2010), not all subfamilies or tribes are represented by exemplars, nor are all genera included (except for the Plumariidae), and the characters used did not necessarily include all those which have been found useful within all of the families, so these results are incomplete in that respect; the aim of including the exemplars used was to reflect the variation found within the families rather than to discover intra-family relationships (except for Plumariidae).

*Plumalexius* seems convincingly indicated as sister to the Plumariidae, although one analysis was ambiguous about this; trees with it placed as sister to the remaining chrysidoids or as sister to the Chrysidoidea as a whole differ in length from that shown in Figure 22 by only 4 and 5 steps respectively (lengths 478 and 479 compared with 474), emphasising its relatively basal position. It does not share any unique synapomorphies with Plumariidae, however (the five unambiguous states supporting the sister relationship to Plumariidae are 11-2: flagellomere setae conspicuous and erect; 21-1: pronotal posteroventral margin strongly concave; 45-1: metasternum weakly depressed anteromedially; 50-0: pterostigma large and prominent; and 87-0: hypopygium completely exposed or almost so, all states found elsewhere in relatively distantly related taxa). The long erect flagellar setae of *Plumalexius* and some Plumariidae have been indicated as a putative synapomorphy for the family (Rasnitsyn 2002: Fig. 331). The arrangement of the setae in *Plumalexius* is most similar to that in *Myrmecopterina*, although the setae are less dense and considerably longer in *Plumalexius*, but the present analysis has shown that other plumariid genera lack such setae and, conversely, they are also present in some Scolebythidae and Bethylidae at least; prominent flagellomere setae are actually found widely in the Chrysidoidea. The other most obvious similarity, more extensive venation in both wings than in other chrysidoids, is a symplesiomorphy. It is thus evident that there is no key apomorphy associating *Plumalexius* with the Plumariidae sufficient to assign it to that family. Were that to be done, the expanded family would lose its present defining features, such as the presence of apical accessory veins in the wing membrane, the reduced second submarginal cell and the tapered mandibles with few apical teeth. In view of this, I conclude that the best solution is to propose a new family for it, as has been done above, something which also emphasises its distinctiveness. In contrast to the specialised morphology of Scolebythidae, showing several adaptations enabling the effective parasitisation of wood-boring beetle larvae, the morphology of *Plumalexius* provides little clue as to its biology, specially since the female is unknown. The male looks like a very generalised wasp, probably very similar to the form ancestral to Chrysidoidea as a whole.

Apart from the above results, the variety of analyses performed has shown that the use only of an outgroup which is sister to the ingroup, and which may have many characters with relatively more-derived states than the ingroup, may produce misleading or ambiguous results (Figs 14–17 all show different relationships from the preferred result). Instead, the outgroup should be expanded to include taxa similarly related to both the ingroup and its sister group. Furthermore, the use of additive characters where reasonable inferences of additivity can be made is likely to produce better-resolved cladograms than if all characters are considered non-additive, and it seems that using implied weighting not only improves the results obtained under both scenarios, but also reduces the uncertainty induced by considering all characters non-additive. The results obtained here, therefore, indicate that wherever possible additive characters and a method (such as implied weighting) which gives greater weight to the more reliable characters should be used.

Whether *Plumalexius* is sister to Plumariidae or not affects the estimated minimum age of Plumariidae: if it is, then Rasnitsyn’s (2002, 2010) estimate remains reasonable (after all, the common ancestor of two lineages must be at least as old as either lineage), but if it is sister to the remaining chrysidoids or to Chrysidoidea as a whole, then that estimate for Plumariidae is poorly founded. Since all other chrysidoid lineages date from the Early Cretaceous (Engel and Grimaldi 2006; Rasnitsyn 2010), that would also be the estimated minimum age for Plumariidae itself if it is considered to be sister to the other chrysidoids rather than to *Plumalexius*. In any case, the presence of a group apparently closely related to Plumariidae in North America in the Cretaceous requires reassessment of ideas on the geographic origin of Plumariidae, making it unlikely that the group arose on Gondwanaland. Like the Scolebythidae, it is probable that the modern members are scattered relicts of a group with a previously much more extensive distribution. The discovery of fossils clearly attributable to Plumariidae will be critical in solving the puzzle, but such fossils are not very likely to be found if members of the family have always been adapted to arid environments, probably being parasitoids of subterranean hosts (Evans 1967), most likely beetle larvae. In contrast, Scolebythidae tend to be found in wooded or forest habitats as parasitoids of wood-boring beetle larvae, and thus have often been entombed in exuded resin and become inclusions in amber, facilitating their later discovery. Both *Plumalexius* and *Boreobythus* Engel and Grimaldi 2007 (Scolebythidae) were found in the same amber deposits, putatively derived from temperate coastal or deltaic swamps of coniferous trees (Grimaldi, Shedrinsky and Wampler 2000), an environment very different from those where modern Plumariidae exist.

## Supplementary Material

XML Treatment for Plumalexiidae

XML Treatment for Plumalexius

XML Treatment for Plumalexius rasnitsyni

## Appendix A. Specimens (all males) utilised for derivation of Table 1

All specimens are in D.J. Brothers’ collection (to be deposited in Iziko South African Museum, Cape Town (SAM), in due course) unless otherwise stated.

Ichneumonoidea:

Ichneumonidae: *Cryptinae* sp. (S. Africa), Pimplinae spp. (2, Malawi, S. Africa)

Trigonaloidea:

Trigonalidae: *Taeniogonalos maculata* (Smith), *Trigonalys* ?*micanticeps* (Strand)

Evanioidea:

Evaniidae: *Evania* sp. (S. Africa), *Acanthinevania* sp. (Australia)

Gasteruptiidae: *Gasteruption* spp. (2, Botswana, S. Africa)

Vespoidea:

Tiphiidae, Anthoboscinae: *Anthobosca* spp. (2, S. Africa)

Sierolomorphidae: *Sierolomorpha bicolor* Evans, *Sierolomorpha canadensis* (Provancher)

Apoidea:

Ampulicidae: *Ampulex* spp. (2, S. Africa), *Dolichurus carbonarius* Smith

Heterogynaidae: *Heterogyna protea* Nagy, *Heterogyna nocticola* Ohl, *Heterogyna madecassa* Day

Chrysidoidea:

Plumalexiidae: *Plumalexius rasnitsyni* sp. nov. (AMNH)

Plumariidae: *Plumarius striaticeps* (André), *Plumarius* spp. (2, Argentina); *Plumaroides andalgalensis* Brothers, *Plumaroides brothersi* Diez and Roig-Alsina; *Myrmecopterina* spp. (3, Botswana, Namibia, S. Africa); *Myrmecopterinella* sp. (S. Africa) (SAM); *Maplurius spatulifer* Roig-Alsina; *Mapluroides ogloblini* Diez, Hidalgo and Roig-Alsina; *Pluroides porteri* Diez, Roig-Alsina and Hidalgo

Scolebythidae: *Scolebythus madecassus* Evans; *Pristapenesia primaeva* Brues, *Pristapenesia inopinata* (Prentice and Poinar in Prentice, Poinar and Milki); *Ycaploca evansi* Nagy

Bethylidae: Mesitiinae: *Pilomesitius* ?*madagascarensis* Moczar, *Sulcomesitius* ?*pondo* (Benoit), *Sulcomesitius* ?*schoutedeni* (Benoit); Epyrinae: *Epyris* spp. (2, S. Africa); Pristocerinae: *Acrepyris fraterna* (Evans), ?*Pristocera* sp. (S. Africa), *Apenesia* sp. (S. Africa)

Chrysididae: Cleptinae: *Cleptes alienus* Patton; Amiseginae: *Amisega similis* Kimsey, *Amisega flavicrus* Kimsey, *Bupon pashoanus* Kimsey; Chrysidinae: Elampini: *Hedychrum nobile* (Scopoli), *Hedychrum* sp. (S. Africa); Allocoeliini: *Allocoelia capensis* (Smith); Parnopini: *Parnopes fischeri* Spinola, *Parnopes edwardsii* (Cresson); Chrysidini: *Chrysis ignita* Linnaeus, *Chrysura pacifica* (Say), *Stilbum cyanurum* (Förster)

Sclerogibbidae: *Sclerogibba africana* Kieffer, *Sclerogibba berlandi* Benoit, *Sclerogibba*?*magrettii* (Kieffer), *Probethylus schwarzi* Ashmead

Dryinidae: Gonatopodinae spp. (2, S. Africa); Dryininae spp. (2, S. Africa)

Embolemidae: *Embolemus collinsi* (Olmi), *Embolemus confusa* (Ashmead), *Embolemus magna* (Olmi), *Embolemus* sp. (New Caledonia), *Embolemus brothersi* Olmi

## Appendix B Characters used for analysis of relationships of Chrysidoidea, all (except last) applicable to males only, treated as additive except where noted otherwise

1. Compound eye, inner margin: sinuate = 0, convex = 1;

2. Compound eye, pores and setae: no pores or setae = 0, scattered pores and/or setae = 1, dense pores and/or setae = 2;

3. Compound eye, setae: absent = 0, minute = 1, short = 2, long = 3;

4. Antennal socket, distance from epistomal suture: less than socket width = 0, more than 1.5 × socket width = 1;

5. Clypeus, shape: transverse, without prominent median lobe = 0; with long median lobe narrower than intermandibular area = 1;

6. Clypeus, form: platelike, apical margin not deflexed = 0, convex, thickened, apical margin deflexed medially = 1;

7. Occipital carina: present = 0, absent = 1;

8. Antenna, antennomere number: more than fourteen = 0, thirteen = 1, twelve = 2, ten = 3;

9. Antenna, radicle-scape axis and insertion: axis nearly straight, simple annular constriction = 0, axis angled, simple annular constriction = 1, axis angled, radicle under flangelike expansion of scape = 2;

10. Antenna, scape form: simple, more or less cylindrical = 0, basally expanded ventromesally = 1, apically expanded ventromesally = 2 (NON-ADDITIVE);

11. Antenna, flagellomere setae orientation: inconspicuous and decumbent = 0, conspicuous and semi-decumbent, some erect = 1, conspicuous and erect = 2;

12. Antenna, flagellomere setae distribution: evenly developed, scattered = 0, better developed ventrally, in irregular transverse rows = 1;

13. Antenna, flagellomere setae length: much less than flagellomere width = 0, about 0.5–1 × flagellomere width = 1, about 1.5–2 × flagellomere width = 2, about 3–4 × flagellomere width = 3;

14. Mandible, form: apical margin truncate (chewing type) = 0, apical margin strongly tapering (cutting type) = 1;

15. Mandible, apical teeth: four or more = 0, three = 1, two or fewer = 2;

16. Maxillary palp, segments: six = 0, five = 1, four = 2, three = 3, one = 4;

17. Labial palp, segments: four = 0, three = 1, two = 2, one = 3, absent = 4;

18. Pronotum, anterior collar (flange): present, covering propleura = 0, present but reduced and exposing propleura = 1, extremely reduced to slight ridge, effectively absent = 2;

19. Pronotum, posterolateral lobe: simple = 0, with preapical vertical blunt carina, posteriorly depressed = 1, with preapical vertical lamella, posteriorly concave = 2;

20. Pronotum, posteroventral angle: rounded = 0, narrowly acute = 1;

21. Pronotum, posteroventral margin: straightish = 0, strongly concave = 1;

22. Propleura, dorsally: separated by membranous region = 0, closely approximated although not fused = 1;

23. Propleura, posterior margin: almost straight, propleura almost entirely contiguous mesad = 0, indented on medial halves, propleura partially separated = 1, mostly indented, propleura almost entirely separated = 2;

24. Proepimeron: clearly distinguishable at outer angle and along posterior margin = 0, reduced, discernible only at outer angle = 1, indistinguishable = 2;

25. Prosternum, form: mostly in a single plane = 0, distinctly biplanar, mostly depressed posteriorly = 1;

26. Prosternum, ventral view: well developed with apophyseal pit(s) = 0, visible as triangular sclerite without apophyseal pit = 1, reduced, scarcely visible = 2;

27. Procoxae, contiguity: contiguous basally = 0, well separated basally = 1;

28. Prepectus, form: very well developed, long (nearing midline) and broad = 0, well developed, short (far from midline) and broad = 1, reduced, short (far from midline) and narrow = 2;

29. Prepectus, midventrally: halves divided, free from each other = 0, halves fused midventrally = 1;

30. Prepectus, fusion: entirely articulating or surrounded by membrane = 0, fused with mesopleuron = 1, fused with pronotum = 2 (NON-ADDITIVE);

31. Notauli: diverging anteriorly = 0, parallel = 1, absent = 2 (NON-ADDITIVE);

32. Scutellum: simple = 0, produced posterodorsally as sharp-edged flange = 1;

33. Mesepimeron, development: well developed, flangelike, overlapping metepisternum = 0, much reduced, abutting metepisternum = 1;

34. Mesosternum, form posteriorly: smoothly truncate = 0, mesad with short transverse carina or weak tooth = 1, mesad with lamella projecting over mesocoxal base = 2;

35. Mesocoxae, contiguity: broadly separated = 0, slightly separated = 1, contiguous = 2;

36. Mesosoma, second dorsal phragma: strongly oblique, muscles 2ph-3ph anterior = 0, scarcely oblique posteriorly, muscles 2ph-3ph anterior = 1, scarcely oblique posteriorly, muscles 2ph-3ph posterior = 2 (NON-ADDITIVE);

37. Metanotum, shape: mesally about as long as laterally or longer = 0, mesally half as long as laterally or shorter = 1;

38. Metanotum, lateral length: less than half medial length of scutellum = 0, more than two-thirds medial length of scutellum = 1;

39. Metapostnotum, development: distinctly lengthened laterally = 0, evenly long throughout = 1, distinctly shortened laterally = 2, shortened throughout = 3, greatly expanded posteromedially = 4 (NON-ADDITIVE);

40. Metapostnotum, hind margin: entirely distinct = 0, distinct medially, indistinct laterally = 1, obliterated medially, indistinct laterally = 2, entirely obliterated = 3 (NON-ADDITIVE);

41. Mesosoma, third dorsal phragma: forming distinct even flange = 0, forming narrow even flange = 1, forming narrow even flange laterally only = 2, medially reduced but distinguishable = 3, absent medially = 4, entirely absent = 5 (NON-ADDITIVE);

42. Metapleuron, endophragmal pit and sulcus: pit posteriorly placed, sulcus angled or rounded ventral to pit = 0, pit posteriorly placed, sulcus acutely extended ventral to pit = 1, pit posteriorly placed, sulcus entirely dorsal to pit and curved = 2, pit anteriorly placed, sulcus entirely dorsal to pit and straight = 3, pit anteriorly placed, sulcus acutely extended ventral to pit = 4 (NON-ADDITIVE);

43. Metathoracic-propodeal pleural suture, dorsally: distinct and complete = 0, reduced but partly discernible = 1, obliterated = 2;

44. Metathoracic-propodeal pleural suture, ventrally: distinct and complete = 0, reduced but partly discernible = 1, obliterated = 2;

45. Metasternum, form anteromedially: not depressed = 0, weakly depressed = 1, entirely depressed = 2;

46. Metacoxal cavities: open = 0, closed = 1;

47. Propodeum, length: longer than high = 0, shorter than high = 1;

48. Propodeum, declivity: imperceptibly merging with disk, not identifiable = 0, distinct from disk = 1;

49. Forewing, tubular/nebulous veins: reaching apical margin = 0, ending before apical margin = 1;

50. Forewing, pterostigma: large and prominent = 0, medium to small but distinct = 1, very small, indistinct or absent = 2;

51. Forewing, closed cells and cell 2Cu (variable 1): ten (C, R, 1Cu, 1R1, 1M, 2Cu, 2R1, 1Rs, 2M, 2Rs) = 0, seven (C, R, 1Cu, 1R1+1Rs, 1M, 2Cu, 2R1) = 1, seven (C, R, 1Cu, 1R1, 1M, 2Cu, 2R1)= 2, six (C, R, 1Cu, 1R1, 2Cu, 2R1) = 3, seven (R, 1Cu, 1R1+1M+1Rs, 2Cu, 2R1, 2Rs, 2M) = 4, six (R, 1Cu, 1R1+1M+1Rs, 2Cu, 2R1, 2M) = 5, seven or fewer (cells 2Cu, 2M and 2Rs open or lost) = 6 (NON-ADDITIVE);

52. Forewing, closed cells and cell 2Cu (variable 2): six or more (2Cu present and closed) = 0, seven (C, R, 1Cu, 1R1, 1M, 2R1, 1Rs) = 1, six (C, R, 1Cu, 1R1, 1M, 2R1) = 2, five (C, R, 1Cu, 1R1, 2R1) = 3, four (C, R, 1Cu, 1M) = 4, three (C, R, 1Cu) = 5;

53. Forewing, costal cell: broad, wider than thickness of bounding veins = 0, narrow, as wide as thickness of bounding veins or less = 1, eliminated, veins C and Sc+R+RS fused = 2;

54. Forewing, 2nd submarginal cell (1Rs) shape (absent coded “-”): anteriorly broadly sessile = 0, shortened and anteriorly briefly sessile to briefly petiolate = 1, much reduced and anteriorly strongly petiolate = 2;

55. Forewing, marginal cell anterior margin: more than 0.7 × pterostigma width = 0, less than 0.5 × pterostigma width = 1;

56. Forewing, apical accessory vein (“RS2”): absent, not even spectral = 0, present, nebulous or spectral = 1;

57. Forewing, prestigma (vein Sc+R) form: narrow, apically less than 1.5 × width of 1Sc+R = 0, broad, apically more than 1.8 × width of 1Sc+R = 1;

58. Forewing, prestigma (vein Sc+R) length: at least as long as 1RS = 0, less than 0.5 X length of 1RS = 1;

59. Forewing, vein Cu2: present = 0, absent = 1;

60. Hind wing, basal hamuli: several, dispersed along costal margin = 0, few, concentrated into cluster = 1, one = 2, absent = 3;

61. Hind wing, tubular/nebulous veins: reaching apical margin = 0, into apical half of wing but not reaching apical margin = 1, restricted to basal half of membrane = 2;

62. Hind wing, veins C and Sc+R: both long and separated, cell C closed = 0, both long and fused = 1, C short, Sc+R absent except at base = 2, C short but distinct, Sc+R long = 3, C absent except at base, Sc+R long = 4, C absent except at base, Sc+R short = 5 (NON-ADDITIVE);

63. Hind wing, vein M+Cu: well developed, tubular = 0, distinguishable but nebulous = 1, absent = 2;

64. Hind wing, anal veins: A1 well developed, A2 present = 0, A1 well developed (more than half length anal lobe), A2 absent = 1, A1 short (less than half length anal lobe), A2 absent = 2, A1 minute, A2 absent = 3;

65. Hind wing, veins rs-m and cu-a: both present = 0, both absent = 1;

66. Hind wing, incised anal (vannal/plical) lobe: absent, at most indicated by slight notch = 0, present, distinct acute incision = 1;

67. Hind wing, anal (vannal/plical) lobe length: short, less than 0.5 × length of wing = 0, long, more than 0.6 × length of wing = 1;

68. Hind wing, jugal lobe: present, delimited apically by incision = 0, absent = 1;

69. Tarsal claws, form ventrally: toothed = 0, simple = 1;

70. Protrochanter, basal insertion: apical on coxa = 0, basolateral on coxa = 1;

71. Foretarsus, arolium: narrower than tarsal apex and shorter than claws = 0, at least as broad as tarsal apex and at least as long as claws = 1;

72. Meso- and metatarsi, arolia: well developed = 0, vestigial = 1;

73. Mesocoxa, subdivision and insertion: large basi- and disticoxites, cavities large = 0, reduced basicoxite and large disticoxite, cavities moderate = 1, much-reduced basicoxite and large disticoxite, cavities small = 2;

74. Mesotrochantellus: distinctly present = 0, reduced but discernible as complete ring = 1, much reduced, almost indiscernable, ventrally only = 2, absent = 3;

75. Mesotibia, spurs: two = 0, one = 1, none = 2;

76. Mesotibia, scattered spines: absent = 0, weak = 1, moderately strong = 2;

77. Metacoxa, specialised area of setae on ventral surface: absent = 0, present = 1;

78. Metatrochanter, specialised area of setae on ventral surface: absent = 0, present = 1;

79. Metafemur, apex: simple = 0, with toothlike projection on each side at tibial articulation = 1;

80. Metatibia, inner apical spur: simple, similar to outer spur = 0, forming calcar with weak simple dorsal carina = 1, forming calcar with dorsal setose carina = 2, forming calcar with dorsal setal fringe only = 3, forming calcar with fine dorsal pectination = 4 (NON-ADDITIVE);

81. Metasoma-propodeum attachment: ventral, between hind coxae = 0, dorsal, distant from hind coxae = 1;

82. Metasoma, T1 articulation with S1: overlapping and articulating with base of S1 = 0, narrowed and fused with base of S1 dorsolaterally = 1;

83. Metasoma, T7 surface: simple, ecarinate = 0, longitudinally carinate = 1;

84. Metasoma, T7 apical flange: absent = 0, present, narrow = 1, present, broad, 0.33 × as long as tergum = 2;

85. Metasoma, S1: ecarinate = 0, with strong median longitudinal carina = 1;

86. Metasoma, S7: well developed and exposed = 0, reduced and partly exposed = 1, much reduced and concealed = 2;

87. Metasoma, hypopygium (S8) concealment: completely exposed or almost so = 0, partially concealed = 1, completely concealed or almost so = 2;

88. Metasoma, hypopygium (S8) shape: simple with blunt to rounded apex = 0, triangular with pointed apex = 1, truncate with weakly emarginate apex = 2, peglike = 3 (NON-ADDITIVE);

89. Metasoma, cercus: present, well developed, cylindrical = 0, present but much reduced, cylindrical = 1, absent or vestigial flattened setose disk = 2;

90. Sexual dimorphism, antennomere number: absent = 0, present (♂ 13, ♀ 12) = 1.
